# Role of FGFR3 in Urothelial Carcinoma

**DOI:** 10.30699/IJP.14.2.148

**Published:** 2019-06-10

**Authors:** Malik Akanksha, Sundaram Sandhya

**Affiliations:** 1 *Post graduate, Sri Ramachandra Medical College and Research Institute, Porur, Chennai, India*; 2 *Professor, Department of Pathology, Sri Ramachandra Medical College and Research Institute, Porur, Chennai, India*

**Keywords:** FGFR3 Protein, Urothelial carcinoma, Carcinoma, Transitional Cell, Bladder cancer

## Abstract

**Background and Objective:**

This study was undertaken to analyze the immunohistochemical expression of fibroblast growth factor receptor (FGFR3) in urothelial carcinoma and correlate its expression with the pathological stage, recurrence and other clinicopathological parameters.

**Material and Methods:**

A retrospective study was undertaken on paraffin blocks of 55consecutiveurothelial carcinoma specimens in 28 months received in Sri Ramachandra Medical College, Chennai, India. Blocks with the sections containing the tumor and adjacent normal epithelium were chosen for the immunohistochemical (IHC) study of FGFR3.

**Results:**

IHC expression of FGFR3 in high grade (HG) invasive urothelial carcinoma was positive in 18% cases, 66.7% of HG non-invasive urothelial and 82.6% of low grade (LG) non-invasive urothelial carcinomas.

The FGFR3 expression was presented in 78.1% of non-invasive carcinoma. In case of invasive urothelial carcinoma, the FGFR3 positivity was observed in 18.2% of tumors (*P*<0.05).

FGFR3 expression in LG tumors was positive in 82.6 % of the cases whereas 32.3% of HG cases were positive for FGFR3 (*P*<0.05).

FGFR3 was expressed in 14.3 % of HG invasive tumors which recurred. HG non-invasive tumors were positive for FGFR3 in 80% of the cases. LG non-invasive tumors were positive for FGFR3 in 72.7% of cases (*P*<0.05).

**Conclusion:**

The expression of FGFR3 is higher in low grade, non-invasive tumors and recurrent non-invasive tumors. The targeted therapy for FGFR3 may be used as one of the modes of treatment for urothelial carcinoma. It can also be used as a marker to determine the grade in difficult cases and the risk of recurrence.

## Introduction

Bladder cancer is the seventh most common malignancy worldwide, accounting for approximately 3.2% of all cancers globally. It is more often seen in males than females. An estimated of 260,000 and 76,000 new cases occur each year in men and women, respectively (1).

Urothelial carcinomas may present as papillary or flat neoplasms, majority of which are usually non-invasive. Diverse molecular pathways are implicated in the pathogenesis and development of non-invasive and invasive tumors (2).

Currently, the depth of invasion, histologic grade and margin status are the most important prognostic factors. However, several other parameters such as p53 expression, ki - 67, loss of E cadherin, CK 20 and FGFR3 mutations have been implicated as indicators of progression and recurrence of the tumor.^1^ Bladder tumors have a high rate of recurrence and progression, thus, prognostic outcome for urothelial carcinoma remains unpredictable, demanding validation of important markers . 

Binding of the fibroblast growth factor receptor (FGFR) to the mutated FGFR3 tyrosine kinase receptor leads to the activation of downstream pathways including RAS- MAPK, PI3K and STAT6. These pathways regulate a number of cellular functions including differentiation and division. Activation of the wild type FGFR3 may occur via ligand independent dimerization of the over-expressed protein, increased expression of ligand or via differential splicing that generates a splice variant such as FGFR3c with the altered ligand specificity (3).

FGFR3 mutations have been described in spermatocytic seminoma, multiple myeloma and cervical cancer. Some cases of multiple myeloma are seen to express both mutation and over-expression of FGFR3. 

Mutations of FGFR3 are found in around 80% of pTa tumors (2,3). *FGFR3 *mutations are present in 21% of pT1 and 16% of pT2–4 tumors (2).

These mutations which occur at the level of genome, are technologically difficult to detect in the routine laboratory (4), therefore, this study was conducted to analyze the immunohistochemical expression of FGFR3 in urothelial carcinoma and correlate the FGFR3 expression with the pathological stage, recurrence and other clinicopathological parameters.

## Materials and Methods

This is a retrospective study on paraffin blocks of 55consecutive urothelial carcinoma specimens received in the Department of Pathology at a tertiary care centre, Sri Ramachandra Medical College, Chennai, India from January 2013 to May 2015. The paraffin blocks were made on samples from tumor areas along with the adjacent normal areas from the specimens received in the department. 

Permission of the institutional ethics committee was obtained prior to commencing the study. Gross findings were recorded and clinical data of the patients including patient age, gender, metastatic status, cystoscopy findings, prognosis and outcome along with the important physiological parameters were obtained from the medical record section and local area computer network service.

The Haematoxylin and Eosin (H&E) stained slides were reviewed by two pathologists and the diagnosis was made as per the WHO 2016 criteria of urothelial tumors.

The staging was done in accordance with the TNM AJCC 8^th^ edition into pTa, pT1 and pT2 tumors.

**Inclusion criteria**

Microscopically proven cases of urothelial carcinomas including low grade non-invasive, high grade non-invasive, low grade invasive and high grade invasive.

**Exclusion criteria**

- Urothelial carcinoma arising from kidney and ureter;
- Bladder malignancies other than urothelial carcinoma.
- Recurrence was considered as cases which had visible tumor on follow up cystoscopy and were confirmed by the subsequent histopathological evaluation.
- pTa tumors (non-invading lamina propria) and pT1 tumors (invading into lamina propria) were categorized as non-muscle-invasive tumors and pT2(invading detrusor muscle) were categorized as muscle-invasive tumors due to the difference in different treatment modalities.

The non-muscle-invasive tumors were treated with chemotherapy and intravesical BCG therapy. One case of LG non-invasive urothelial carcinoma underwent radical cystectomy due to failure of BCG therapy.

The cases with muscle invasion underwent radical cystectomy, however, the patients who were found to be unfit for the surgery in this category underwent TURBT and chemoradiotherapy.

Paraffin blocks made after routine processing were cut as 3 microns sections and were stained with H & E for the routine morphology. Tumor grading was performed based on the architectural and cytological features. 

Blocks with the section containing the tumor and adjacent normal epithelium were chosen for immunohistochemical study. This Immuno-histochemical study of FGFR3 (Clone: B-9: sc 13121, Subtype: IgG_2a_, Source: mouse, Immunogen: amino acids 25-124 of FGFR3 of human origin, Santa Cruz Biotechnology, USA) was conducted on the estimated sample size of 55 cases of histopathologically proven urothelial carcinoma cases. The IHC was performed on 0.1% poly-L-Lysine coated slides, which were then kept in the incubator for 30 minutes at 60°C. The slides were placed in citrate buffer at pH of 6.0. Heat induced antigen retrieval was done by pressure cooker with an operating pressure of 103kpa/115 at 120°C, then, blocking was done by 3% hydrogen peroxide. The antibody was used in a dilution of 1:50 and incubated overnight.

Previous studies have shown that the human epidermal cells were strongly positive for FGFR3, which was interpreted as positive control^5^.

As negative control, the slide was treated by replacement of primary antibody with non-immune serum (28).

All the slides were examined and scored according to the Q score.

Q score (6):

A semi-quantitative scoring system was adopted: 0, all tumor cells negative; 1, faint but detectable positivity in some or all cells; 2, weak but extensive positivity; 3, strong positivity. Percentage staining score was graded as 0 to 4.

Q score = intensity × percentage staining 

Q score of 0 and 1 were taken as negative result and 2 to 12 as positive result.

FGFR3 expression was observed in the cytoplasm and nucleus^4^. However only one case of low grade non-invasive carcinoma showed nuclear positivity, which was an exception in our study, therefore it was not included in the statistical analysis. 

The statistical analysis was done on the data collected using the “SPSS Version 11” statistical program. Pearson Chi-square test was used to determine significant clinicopathological differences between FGFR3 expression in positive and negative tumors. Differences were considered statistically significant when P-value was < 0.05. 

## Results

**Patients’ characteristics, treatment and recurrence**

The study comprised of 55 patients, of whom low grade non-invasive tumors were diagnosed in 24 (43.6%) ones. However, in our study no low grade invasive tumor was found. High grade invasive tumors accounted for 22 (40%) of all the cases, whereas high grade non-invasive were found to be 9 (16.4%).

The patients were followed up for 5 to 36 months, mean follow-up period was 24 months. In our study, eight HG invasive and one LG non-invasive tumors underwent radical cystectomy. Of the remaining 46 cases, 93.3 % (14/15) HG invasive recurred. Amongst HG non-invasive cases, 62.5% (5/8) recurred. The recurrence was documented in 47.8 % (11/23) of LG non-invasive tumors. 

**FGFR3 expression**

IHC expression of FGFR3 antibody in HG invasive urothelial carcinoma was positive in 18% (4/22) cases. The Q score in HG invasive tumors ranged from 0 to 4, average was 0.72 ±1.20. 

In HG non-invasive urothelial carcinomas the positivity was seen in 66.7% (6/9) of cases. High grade non-invasive tumors had a range of Q score from 1 to 6, with an average of 3.11 ± 1.76.

The highest positivity was seen in LG non-invasive urothelial carcinoma 82.6% (19/23) of tumors. LG non-invasive tumors had a Q score ranging from 1 to 12 with an average of 5.86 ± 4.28. One case of LG non-invasive urothelial carcinoma had nuclear positivity for FGFR3.

**FGFR3 expression with grade and stage**

In non-invasive carcinoma FGFR3 expression was present in 78.1 % (25/32) whereas, it was negative in21.9% (7) of the tumors.

In case of invasive urothelial carcinoma FGFR3 positivity was observed in 18.2% (4/22) of tumors, while 18 or 81.8% were negative.

The P-value was significant (*P*<0.05).

FGFR3 expression in low grade tumors was positive in 82.6 % (19/23) and negative in 17.4% (4/23) of the cases. And 67.7 % (21/31) of high grade tumor cases were found to be negative whereas 32.3% (10/31) were positive for FGFR3. The P-value was found to be significant (*P*<0.05)

**FGFR3 expression in recurred cases**

FGFR3 was expressed in 14.3% (2/14) of HG invasive tumors which recurred. In this category 85.7% of the cases were negative. HG non-invasive tumors were positive in 80% (4/5) of the cases, whereas 20% were negative for FGFR3.

LG non-invasive tumors were positive for FGFR3 in 72.7% (8/11) of the cases, whereas 27.3% (3/11) were negative. 

This was significant with a P-value of <0.05.

**Table 1:** Patient details

**Age at diagnosis**	19-87 years
**Mean age**	61.5 years
**Highest incidence**	60-69 years
**Male : female**	4:1
**Cigarette smoking**	30 (54.5%)
**Hematuria**	46 (86.6%)
**Urinary tract infection**	26(47.3%)
**Pain**	30(54.5%)
Cystoscopy findings	
**Single**	20 (36.4%)
**Multiple**	35(63.6%)
Histopathological diagnosis	
**Low grade non-invasive urothelial carcinoma**	24
**Low grade invasive urothelial carcinoma**	0
**High grade non-invasive urothelial carcinoma**	9
**High grade invasive urothelial carcinoma**	22

**Table 2:** FGFR3 positivity with grade and stage

	LG non-inv UCA	HG non-inv UCA	HG inv UCA	Low grade UCA	High grade UCA	P-value	Non-inv UCA		Inv UCA	P-value
FGFR3 positivity	19/23 (82.6%)	6/9 (66.7%)	4/22 (18%)	19/23 (82.6%)	10/31 (32.3%)	<0.05	25/32 (78.1)	4/22 (18.2%)		<0.05

**Table 3:** FGFR3 positivity in recurred cases

	LG non-inv UCA	HG non-inv UCA	HG-inv UCA	P-value
FGFR3 positivity	8/11 (72.7%)	4/5 (80% )	2/14 (14.3 %)	<0.05

LG non-inv UCA: low grade non-invasive urothelial carcinoma

HG non-inv UCA: high grade non-invasive urothelial carcinoma

UCA : urothelial carcinoma

## Discussion

In our study on56 patients, the male to female ratio was 4:1, which corresponded to the studies done before (7,8). The mean age was 61.5 which was in concordance with a large study done on the Indian population comprising of 561 patients, in which the mean age was 60.5 (11). In our study, the youngest patient had19 years old and presented with LG non-invasive urothelial carcinoma with no recurrence. In young patients there is higher frequency of low grade tumors, and the disease at the time of diagnosis is more frequently at a low stage^9^. In a study conducted on 45 patients younger than 30 years Huang et al., observed that patients aged 25 years or younger were less likely to experience tumor recurrence and expressed more proportion of the negative pattern of FGFR3 protein (10). In the current study the patient did not have a recurrence and expressed low level of FGFR3, with a Q score of 2.

The history of smoking was present in 54.2% of cases. The risk of bladder cancer is 2-6 times in smokers than in no- smokers. In two Indian population-based studies the incidence of smokers with bladder cancer were found 68.6 % and 71.6% (11, 12). However, in our study, slight discrepancy may be due to incomplete history by non-compliant patients or a natural outcome of our study population.

Spruck et al. proposed that cigarette smoke does not significantly change the type of mutations of p53 in smokers and non-smokers; however, it makes the DNA more susceptible to damage (13).

The most common clinical presentation in our study was hematuria accounted for 86.6% of the initial symptoms. This is in agreement with the reported 80 to 85% in world literature (1).

The 54.5% patients presented with pain as a presenting complaint, while, urinary tract infection accounted for 47.3% of the clinical presentations.

As examined cystoscopically, 63.6% and 36.4% of the tumors were multiple and single, respectively. Tumor multifocality and concurrent carcinoma in situ have been identified as risk factors for the recurrence and progression (1).

The most common histological subtype was low grade non-invasive accounting for 43.6%. High grade non-invasive were found to be 16.4%. Hence, non-invasive tumors accounted for 58.9%.

High grade invasive tumors accounted for 40% (22) of all the cases. There were no instances of low grade invasive tumors in our study. 

According to a study by Gupta et al., on Indian population, 26% of the patients had muscle-invasive disease at the time of presentation while the remaining 74% had non-muscle- invasive bladder carcinoma (11).

A study by Tomilson et al., found a highly significant association between the protein expression level and mutation status.^3^Here, in our study we compared the FGFR status with grade and stage. Young-Hee Maeng et al. reported 78.9% of pTa tumors expressed FGFR3 by immunohistochemistry (4). Tomilson et al., on IHC reported 81% positivity in non-invasive tumors (3). In our study, 78.1% of non-invasive carcinomas expressed FGFR3shown by IHC. Gomez roman reported IHC expression of 71.4 % of non-invasive tumors (14).

In a study done by Poyet et al.in 2015 a positivity of 69% for pTa tumors was reported (15).

However, Matsumoto et al., did not report any significant association between FGFR3 expression and stage of tumor (16).

In the present study there was a significant association between FGFR3 expression and the stage (*P*< 0.05).

In our study, the FGFR3 expression in high grade invasive tumors (pT2 – pT4) was found to be 18.2% of cases. This showed a significant association between the stage and FGFR3 expression with P-value< 0.05. Our value was in concordance with a study by Bodoor et al (17) on 130 patients who got the FGFR3 expression in 15% of pT2 and 2% of pT3 tumors. However, they did not find any significant association between the stage and the expression level, which could be due to tumor heterogeneity.

Guancial et al., in 2015 studied 231 primary muscle-invasive tumors and found a positivity of 29% by IHC (18). On the other hand, Gomez Roman observed 49% positivity in pT2 tumors (14).

In our study, positivity and negativity for the low grade tumors was assessed to be 82.6% and 17.4% cases, respectively. The 81.3% of low grade tumors were positive as reported by a Korean study (4). According to the study done by Poyet et al., in the low grade tumor group, 69% of tumors were FGFR3 positive (15).

Young-HeeMaeng et al., reported 47.8% of high grade tumors to be positive for FGFR3 (4). In the present study, we found 32.3% of high grade tumors to be positive.

In an Iranian study, the authors found an increased expression of FGFR3 in most of the samples inall grades and stages (19).

The association of FGFR3 with grade was significant in our study with a P-value <0.05.

Non-invasive high grade tumors recur frequently in up to 70% of cases, but progression is rare (1,20). High grade non-invasive tumors recur frequently and progress in up to 65% of cases (1).

The role of FGFR3 in predicting the prognosis and progression remains unclear. Mutation studies done on two groups of the same have reported an association between mutations and a higher recurrence rate (Mhawech-Fauceglia P et al, Van Oers et al) (21,22, 23) , whereas others are contrary (van Rhijn BW et al, Hernández S et al) (24,25). Few studies have been done by immunohistochemistry. Young-Hee Maeng et al. reported FGFR3 to be of prognostic value for recurrence-free survival in non-muscle-invasive tumors (4), although it was not an independent marker.

Poyet et al., in 2015 reported that patients with tumors expressing FGFR3 confirmed by IHC had a significantly increased disease specific survival as compared to the negative expression. High grade neoplasms in their study showed a trend of better disease specific survival but it was not statistically significant (15).

On the other hand, Bodoor et al. did not find any prognostic value of FGFR3 for recurrence free survival, although it was not an independent marker (17).

Low grade non-invasive tumors which recurred were positive for FGFR3 in 72.7% of the cases. High grade non-invasive tumors were positive in 80% of the cases.

In the current study, invasive tumors which recurred were positive for FGFR3 in 14.3% of the cases. This was found to be statistically significant (*P *< 0.05).

In the cases of high grade invasive carcinoma which underwent radical cystectomy only one out of nine cases showed positivity for FGFR3.

Guancial et al., did not find any association between muscle-invasive urothelial carcinoma and overall survival (18). Turo et al., also reported similar findings (26).

One of the cases in our study was diagnosed to have low grade non-invasive carcinoma who underwent radical cystectomy. Clinically and radiologically he had extensive involvement of the bladder mucosa by papillary neoplasm, with histopathological extension into the prostatic ducts. This case had an interesting FGFR3 staining pattern of nuclear positivity.

Nuclear positivity of FGFR3 has not been studied extensively due to the small proportion of such staining in other studies. In the study done by Rotterud et al., in 2007 (27), they concluded that nuclear positivity of FGFR3 led to the tumor genesis. In a Korean study done in 2010, nuclear FGFR3 positivity was reported in 32.7% of the tumors but it failed to show prognostic power ( 4).

## Conclusion

The FGFR3 expression is seen in urothelial carcinoma. It is high in low grade, non-invasive tumors, and tumors showing recurrences which are non-invasive. The targeted therapy for FGFR3 may be used as one of the modes of treatment for urothelial carcinoma. It can also be used as a marker to determine the grade in difficult cases and the risk of recurrence.
